# Astragalin from *Cassia alata* Induces DNA Adducts *in Vitro* and Repairable DNA Damage in the Yeast *Saccharomyces cerevisiae*

**DOI:** 10.3390/ijms13032846

**Published:** 2012-03-05

**Authors:** Samuel Saito, Givaldo Silva, Regineide Xavier Santos, Grace Gosmann, Cristina Pungartnik, Martin Brendel

**Affiliations:** 1 Laboratório de Biologia de Fungos, Centro de Biotecnologia e Genética, Universidade Estadual de Santa Cruz, Ilhéus, BA, 45662-900, Brazil; E-Mails: samuelsaito@hotmail.com (S.S.); gil_ssg@hotmail.com (G.S.); sxneide@gmail.com (R.X.S.); martinbrendel@yahoo.com.br (M.B.); 2 Faculdade de Farmácia, Universidade Federal do Rio Grande do Sul, Porto Alegre, RS, 90610-000, Brazil; E-Mail: grace.gosmann@ufrgs.br

**Keywords:** *Cassia alata*, astragalin, DNA binding, FTIR, *Saccharomyces cerevisiae*, antioxidant

## Abstract

Reverse phase-solid phase extraction from *Cassia alata* leaves (CaRP) was used to obtain a refined extract. Higher than wild-type sensitivity to CaRP was exhibited by 16 haploid *Saccharomyces cerevisiae* mutants with defects in DNA repair and membrane transport. CaRP had a strong DPPH free radical scavenging activity with an IC_50_ value of 2.27 μg mL^−1^ and showed no pro-oxidant activity in yeast. CaRP compounds were separated by HPLC and the three major components were shown to bind to DNA *in vitro.* The major HPLC peak was identified as kampferol-3-*O*-β-d-glucoside (astragalin), which showed high affinity to DNA as seen by HPLC-UV measurement after using centrifugal ultrafiltration of astragalin-DNA mixtures. Astragalin-DNA interaction was further studied by spectroscopic methods and its interaction with DNA was evaluated using solid-state FTIR. These and computational (*in silico*) docking studies revealed that astragalin-DNA binding occurs through interaction with G-C base pairs, possibly by intercalation stabilized by H-bond formation.

## 1. Introduction

*Cassia alata* L. (Fabaceae) is used in traditional medicine mainly in the tropical areas of the world, such as Malaysia, Brazil, and Indonesia. The leaves of *C. alata* are used as an effective treatment against ringworm and also against other skin diseases such as eczema and chronic skin impurities [1]. The main pharmacological activities cited in the literature are: anti-inflammatory, analgesic, laxative, antifungal and antimicrobial [1,2]. *C. alata* leaves contain emodin, kaempferol, aloe-emodin, chrysophanol and isochrysophanol, rhein, ellagitannin, phenolic acid and cassiaxanthone, amongst other substances [1].

**Figure 1:**
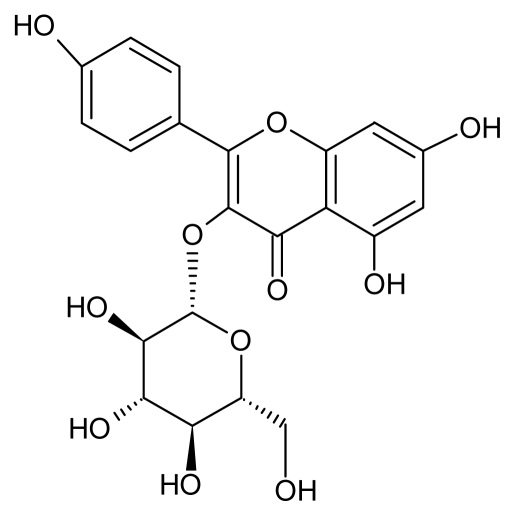
Kaempferol-3-*O*-β-d-glucopyranoside (astragalin).

One of the important flavonoids of *C. alata* leaves is astragalin (AST) (Figure 1). AST is the main constituent of persimmon leaves but has also been found in different plants such as *Eucommia ulmoides*, *Solenostemma argel* and garlic, amongst others [3–6]. AST has lately aroused increased pharmaceutical interest because of its potential as anti-inflammatory agent, in addition to having antimicrobial activity with a MIC_50_ of 83 μg/mL against *S. aureus* (MRSA) [2,3] and also as an alternative medicine for allergic diseases [7].

Studies on molecular interaction of AST with some cell constituents are few and recent. An increase in activity of enzymes of red blood cell membranes (Na^+^K^+^ATPase, Ca^2+^ ATPase, Mg^2+^ ATPase and total ATPases) was observed on lead acetate-induced intoxication co-treated with AST (20 mg/kg per wt/day, 90 days) as compared to those of AST-untreated intoxicated rats [8]. AST induces proliferation of osteoblastic cells but does not have an antiestrogenic effect [6].

Deng and co-workers [9] isolated AST from *Lotus* leaf and showed its interaction with DNA *in vitro*, using fluorescence and ultraviolet absorption spectroscopy. However, detailed information on the mode of AST-DNA interaction and the type of adducts formed with nucleic acids is still not well understood and hence merits further in-depth analysis. The rareness of AST in nature has prevented its widespread study and biotechnological application and only very recently the successful synthesis of AST by enzymatic hydrolysis of tea seed extract has opened a new source for its production [3].

Since AST is known to be present in *C. alata*, the aim of this work was to extract a relative high concentration of the flavonoid from this plant for a possible use in alternative medicine. We first evaluated the antioxidant capacity of the RP-SPE extract from *C. alata* and determined the probable mechanism of action using *in vitro* interaction of CaRP with the eukaryotic model cell *Saccharomyces cerevisiae*. Identification of AST as one of the major *C. alata* metabolites was achieved by applying Fourier transform infra-red spectroscopy (FTIR), high-resolution mass spectrometry (ESI-qTOF-MS/MS) and H^1^ and C^13^ NMR analysis. An in- depth study of AST interaction with DNA was performed using FTIR spectroscopy and *in silico* docking studies. To our knowledge this is the first report that shows *C. alata* extract/compound in interaction with nucleic acids using spectroscopic methods and *Saccharomyces cerevisiae* null mutants as a model of study.

## 2. Results and Discussion

### 2.1. Antioxidant Activity of CaRP

Antioxidant and anti-infectious properties of *C. alata* extracts are well documented in the literature [1,2,10,11]. In order to increase the relative amount of probable bioactive compounds of the aqueous extract of *C. alata* leaves, we decided to refine the aqueous extract using Reverse Phase (C18)-Solid Phase Extraction obtaining a 17-fold concentrated extract named CaRP that showed much more intensive bands in TLC than the original aqueous extract (data not shown). CaRP also presented antimicrobial activity against *Staphylococcus aureus*, *S. epidermidis*, *Bacillus subtilis* and *Pseudomonas aeruginosa* (data not shown). We used the DPPH assay to also search for antioxidant properties of CaRP.

In order to calculate the IC_50_, calibration curves of each standard (ascorbic acid and Trolox) and of CaRP were determined by 2,2-diphenyl-1-picrylhydrazyl (DPPH) assay as described in Material and Methods. The curves proved to be linear in the concentration range shown in Table 1 using linear regression and resulted in a correlation coefficient (*r*) >0.99 to standards and CaRP.

At the conditions used, antioxidant activity of ascorbic acid was higher than that of Trolox. Interestingly, CaRP had higher antioxidant activity than ascorbic acid and Trolox, *i.e.*, 0.5639 g of antioxidant activity of CaRP is equivalent to 1 g of ascorbic acid, or 0.5000 g of CaRP is equivalent to 1 g of Trolox. Compared to the literature, the IC_50_ of CaRP was about 50× less (IC_50_ of CaRP: 2.25 μg/mL; IC_50_ of ethanolic *C. alata* extract: 112.46 μg/mL) than ethanol-extracted without any refined method [11].

### 2.2. Yeast Sensitivity Assay

Since CaRP may be a valuable source of compounds, especially of AST, with its different biological and pharmacological effects, we decided to evaluate the effect of CaRP using *Saccharomyces cerevisiae* mutants lacking synthesis of endogenous antioxidants, DNA repair enzymes or membrane constituents as model cells to find possible targets that could hint at mechanisms of action.

**Figure 2:**
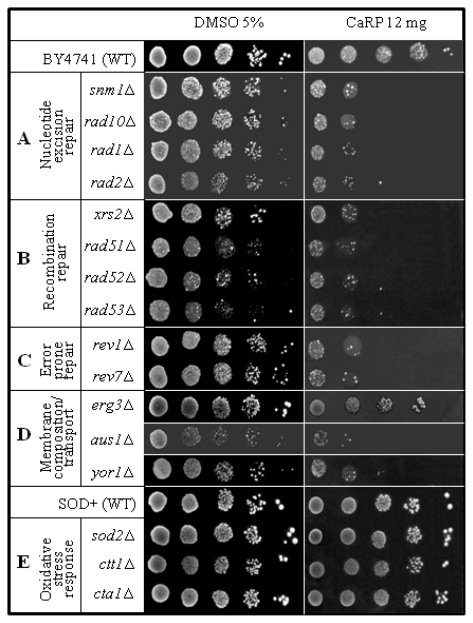
Sensitivity to CaRP of haploid yeast strains.

*S. cerevisiae* null mutants that lack only one gene in comparison to the isogenic wild type offer the possibility to study the relevance of a missing specific metabolic pathway on the response to the defined treatment with the chemical studied. The absence of the protein codified by that gene leads to specific responses described as phenotypes [12] that characterize the metabolic pathway in which it is involved, *i.e.,* mutants in proteins involved in DNA repair are more sensitive to drugs that cause DNA damage that is repaired by that biological pathway. Therefore, this is a very powerful system to rapidly identify a putative mechanism of drug action in a complex cell [13]. Here we used a set of 10 yeast null mutants involved in DNA repair, representing the three so-called epistasis groups, which are thought to reflect three major DNA repair pathways: *RAD3* (involved in nucleotide excision repair [NER], Figure 2A), *RAD52* (recombinational repair, Figure 2B) and *RAD6* (error-prone repair, Figure 2C) epistasis group genes [14–16]; 3 yeast mutants defective in membrane composition or transport across the membrane (Figure 2D); and 3 yeast mutants defective in oxidative stress protection mechanisms (Figure 2E).

Although CaRP (12 mg/plate) did not show any cytotoxic effect in wild type strains (BY4741, *SOD2*), isogenic DNA repair mutants showed higher sensitivity when treated with CaRP (Figure 2A–C). Interestingly, all repair mutants of the three epistasis groups of DNA repair (*RAD3*, *RAD52* and *RAD6* epistasis group) showed some degree of sensitivity. These results suggest that CaRP contains one or more substances that interact with DNA individually or simultaneously in distinct ways, considering that the repair pathway seemed not specific. Additionally, all mutant strains in the *RAD3* (nucleotide excision repair—NER, Figure 2A), *RAD52* (homologous recombination, Figure 2B) and *RAD6* (translesion repair, Figure 2C) epistasis group had similar sensitivity. The higher than wild-type sensitivity to *pso2*Δ (*snm1*Δ) (Figure 2B) suggested that some substance of CaRP might intercalate with DNA and induce lesions that link the two DNA strands. Evidence of this comes from some studies that showed *pso2*Δ mutant specifically blocked in repair of interstrand cross-links [13]. These results could be promising because many antitumoral drugs act by intercalating into DNA and forming interstrand cross-links, e.g., photoactivated psoralens, mitomycin C and *cis*-platinum [17].

Mutants defective in membrane transport such as *aus1*Δ (lacking the function of uptake of exogenous sterols and its subsequent incorporation into the plasma membrane) and *yor1*Δ (lacking multidrug transporter which mediates export of many different organic anions) had the same sensitivity as the DNA repair-mutant strains except for *erg3*Δ (lacking C-5 sterol desaturase) that was almost unaffected, *i.e.*, showed a wild type response (Figure 2D). *Erg3p* is known to protect cells specifically from drugs inducing lipid peroxidation [18]. Lack of sensitivity in *erg3*Δ mutant, therefore, indicates that CaRP did not induce lipid peroxidation.

Conversely, mutants lacking endogenous antioxidants such as superoxide dismutase (*sod2*Δ) or catalase (*ctt1*Δ and *cta1*Δ) had identical response to CaRP as the isogenic wild type that is proficient in all these enzymes (Figure 2E). This result corroborates with the DPPH assay (Table 1) and suggests that CaRP in the applied concentrations is not pro-oxidant in these eukaryotic cells.

### 2.3. Bioactive DNA-Binding Compounds from CaRP

Since CaRP showed cytotoxicity in DNA repair-deficient yeast mutants and in strains with defects in membrane transport, we decided to test if CaRP compounds could bind to DNA *in vitro*. For this, centrifugal ultrafiltration sampling followed by HPLC analysis was used, a fast screening for bioactive compounds binding to DNA [19].

**Figure 3:**
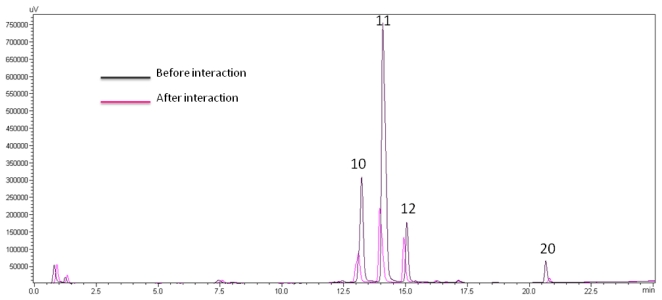
Bio fingerprinting chromatograms for CaRP at 254 nm using 50 μL CaRP (400 μg mL^−1^) (black line) and the mixed CaRP-DNA solution (red line) at a final concentration of 510 μg mL^−1^ in BPES buffer. Peaks are numbered according to the LC-microfractionation.

The Amicom filtrates from CaRP extract and from the mixed solution of CaRP + DNA were collected and analyzed by HPLC (Figure 3). Because the free concentration of ligands decreases in the solution after the interaction (DNA remains in the Amicon membrane and only unbound metabolites with medium polarity can pass through) the peak areas of these compounds decrease in the chromatogram; for those not DNA binding, there should be no or little change in their peak areas before and after the interaction. Therefore, comparing the chromatograms of the Amicon filtrates after the reaction of the control (without DNA) and with DNA, the DNA-binding compounds in the CaRP can be easily distinguished from those that do not have affinity to DNA.

The binding degree of any component to DNA was calculated according to Zhou *et al.* [19]:

$$\text{binding degree}=\frac{A_{\text{b}}-A_{\text{c}}}{A_{\text{b}}}\times 100\%$$

where the peak areas of a compound before (*A_b_*) and after (*A_c_*) the interaction with DNA in the HPLC chromatograms, were measured.

Figure 3 shows the bio fingerprinting chromatograms at 254 nm. The integration of the peak areas showed that 3 peaks in CaRP decreased significantly after interaction with DNA: the respective decreases were 64% for peak 10, 72% for peak 11, and 78% for peak 20.

### 2.4. Structural Identification

We chose to study by FTIR spectroscopy the CaRP fraction equivalent to peak 11 (Figure 3) in its interaction with calf thymus DNA since it is the major fraction (yield of 52%) and also showed cytotoxicity against prokaryotic cells, such as *S. epidermidis* and *s. aureus* [20].

**Figure 4:**
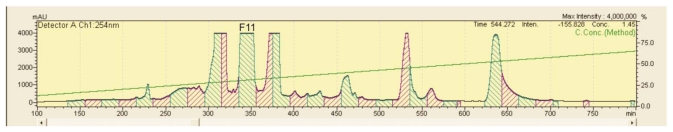
Semi-preparative LC chromatogram of CaRP. F11 is equivalent to peak 11 in the analytical method (HPLC DNA binding).

The equivalent fraction of HPLC peak 11 was obtained by LC-microfractionation (Figure 4). Identification was carried out using FTIR, high-resolution mass spectrometry (ESI-qTOF-MS/MS) and H^1^ and C^13^ NMR.

**Figure 5:**
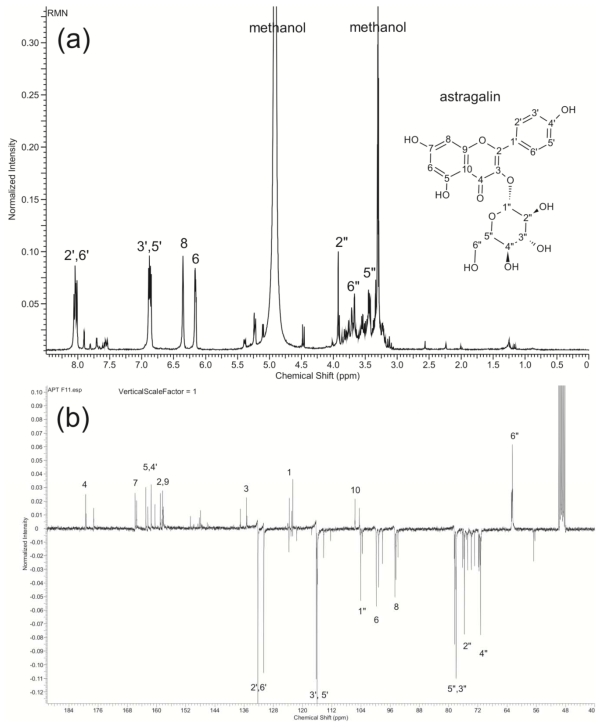
(**a**) ^1^H-NMR and (**b**) ^13^C-NMR (APT) of astragalin in MeOD.

Fraction 11 (F11): yield of 0.523 mg/mg. Green powder, IR (KBr) cm^−1^: 3364 (OH), 1782 (C=O), 1656, 1607, 1505, 1452 (C=C, Ar), 1286 (=C–O–C), 1118 (C–OH); Negative high resolution ESI-qTOF-MS *m/z* 447.1187 [M-H]^−^ (32%), MS-MS fragmentation of *m/z* 447.1187: 284.0699 [M-H-Glu]^−^, 255.0648, 227.0681; H^1^ NMR (MeOD) and C^13^ NMR (MeOD) were carried out and after comparison to data in the literature [3,4], F11 was identified as kaempferol-3-*O*-β-d-glucoside (astragalin) (Figure 5).

Only a little quantity of AST was isolated from various plants such as persimmon leaves, *Eucommia ulmoides* leaves, *Nelumbo nucifera* (Lotus leaves), *Solenostemma argel*, garlic leaf and shoot, *Flaveria bidentis*, *Cuscuta chinensis,* and the seeds of *Centaurea schischkinii* [3–7].

Although this compound has been isolated from *C. alata* leaves before [1,2,21], our results showed that solid phase extraction is an improved technique to obtain the enriched extract CaRP, having also the advantage of an easy and rapid purification method.

### 2.5. FTIR Spectra of AST-DNA Complex

**Figure 6:**
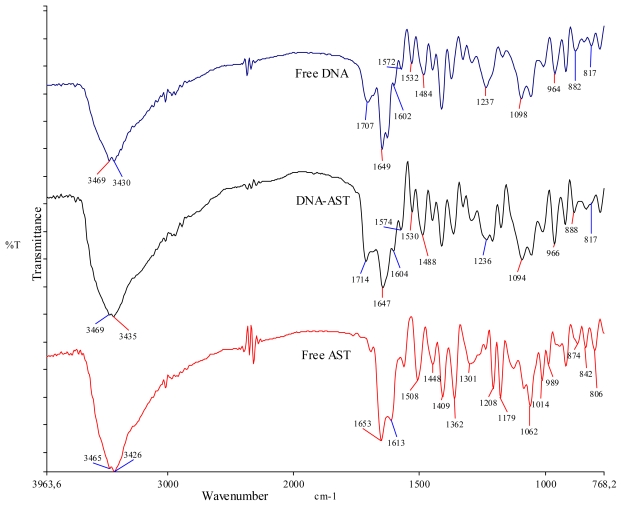
Fourier transform intra-red spectroscopy (FTIR) spectra of free DNA (top), DNA-AST (middle) and free astragalin (AST) (bottom).

The infrared spectrum of AST-DNA mixture offered evidence for the direct binding of this compound to DNA (Figure 6 and Table 2). The vibrational frequencies of NH and OH groups of free DNA appeared around 3469–3430 cm^−1^, which is in accordance to Usha and co-workers [22], and in the AST-DNA complex had a little shift to 3469–3435 cm^−1^. Therefore, the vibrational frequencies of NH band in DNA and OH in the fraction has changed in the complex, evidencing the effective interaction of AST OH with NH of DNA bases, possibly through H-bond interaction.

The vibrational frequency of C=O of the free DNA at 1707 cm^−1^ shifted to 1714 cm^−1^ in the complex. According to Alex and Dupuis [23] further indication for H-bonding interaction of AST with DNA base pairs such G-C and A-T and with phosphate groups comes from major spectral changes of DNA in-plane-vibrations in the region of 1707–1080 cm^−1^ (Figure 6, Table 2). The band at 1707 cm^−1^ (Gua, Thy) related to mainly Gua, 1602 cm^−1^ (Ade, Cyt) related to mainly Ade, and 1484 cm^−1^ (Cyt, Gua) related to mainly Cyt, shifted towards higher frequency with the change in intensity in complex at 1714, 1604 and 1488 cm^−1^, respectively. The band at 1649 cm^−1^ (Thy, Gua, Cyt), mainly for Thy, shifted to lower frequency at 1647 cm^−1^. The observed changes in frequency can be related to AST interaction with Gua, Cyt, Ade and Thy bases. But there is some evidence that AST binds preferentially to Gua beyond the major shift at 1707 cm^−1^ (Gua C6=O stretching) to 1714 cm^−1^, where we can see also the shift of the band at 1572 cm^−1^ (purine N7 stretching) to 1574 cm^−1^ and shift of the band at 1532 cm^−1^ (in-plane vibration of C≡G) to 1530 cm^−1^.

Additional evidence for DNA-AST interaction is obtained from the spectral shifting of AST vibrational frequencies upon DNA binding. Besides the functional OH group of AST, the symmetrical and asymmetrical stretching is also essential in the binding of the aromatic ring to DNA. The frequency vibration of C=C stretching of free AST at 1653 (asymmetrical C=C stretching) and 1613 cm^−1^ (symmetrical C=C stretching) shifted to 1686 and 1628 cm^−1^, respectively. The out-of-plane C–H bending at 806 cm^−1^ in AST shifted to 795 cm^−1^ in the complex. Other minor changes (shifting/intensity variation) were also observed in AST at 1362 cm^−1^ (in plane OH bend), 1208 cm^−1^ (C–O stretching), 1508 and 1448 cm^−1^ (C–C stretching (in ring)) [24].

Minor variation in PO_2_^−^ asymmetric and symmetric vibration at 1237 to 1236 cm^−1^ and 1098 to 1094 cm^−1^, infrared B-marker bands at 882 cm^−1^ (sugar-phosphate stretch) and 817 cm^−1^ (phospho-diester mode) present in free DNA did not appreciably shift in the DNA-AST complex. This result shows that the DNA remains in the B-DNA conformation.

Deng and co-workers [9], using fluorescence and UV-vis absorption spectroscopy analyzed the interaction between *Lotus* leaf derived AST and DNA and computed the binding constant to *K* = 3.41 × 10^4^ M^−1^ and concluded that AST could interact with DNA by intercalative binding. Their data corroborate with ours. By applying FTIR spectroscopy we could obtain the additional information that AST binding to DNA occurs preferentially with Gua-Cyt rather than Ade-Thy base pairs.

It is generally accepted that small molecules might bind to a DNA double helix by three modes: electrostatic binding, groove binding and intercalative binding [25]. Normally, small molecules have to some extent selectivity except for their binding to DNA by electrostatic interaction (along the external DNA double helix) [19]. Intercalative binding is usually affected by planarity of the bound candidates and groove binding is related to the two types of grooves in DNA, *i.e.*, major and minor groove. In fact, each small molecule has a particular structure, which possibly has different binding modes with DNA and each mode resulting in a characteristic distortion of DNA, and this in theory giving rise to a specific pharmacological effect [26]. Indeed, *in vitro* studies of compound-DNA interaction allow valid predictions to the possible *in vivo* interactions with DNA and their cytotoxic/genotoxic consequences (Figure 2).

However, it should be stressed that we have no direct prove that AST reaches nuclear DNA. TOXNET database [27] has so far no entry related either to mutagenicity, genotoxicity nor to carcinogenicity of this compound. Our *S. cerevisiae* data (Figure 2) give some hint that nuclear uptake of CaRP compounds may occur. Whether it is AST or one of the metabolites cannot be answered by our experiments, although the significantly higher sensitivity of yeast DNA repair mutants and the abundance of AST (> 50%) in CaRP suggests this.

### 2.6. Docking Study

**Figure 7:**
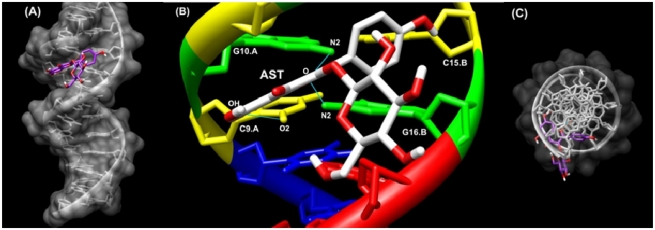
Docking structure between d(CGCGAATTCGCG)_2_ (PDB ID:1D30) and AST. (**A**) Surface representation of d(CGCGAATTCGCG)_2_ complexes with AST (Display sideways); (**B**) Close up view of d(CGCGAATTCGCG)_2_ complexes with AST represented in the stick style. Hydrogen bonds between d(CGCGAATTCGCG)2 and AST were calculated by Chimera software and are drawn in blue lines; (**C**) Surface representation of d(CGCGAATTCGCG)_2_ complexes with AST (Display top).

To determine its preferred binding sites on sequence of DNA [CGCGAATTCGCG (PDB ID:1D30)], AST was docked to DNA using HexServer [28]. The docking result is shown in Figure 7. The model shows that AST is surrounded by C9.A, G10.A, C11.A, C15.B, G16.B and A17.B of the oligonucleotide 1D30 (Figure 7). For the AST-1D30 complex, docking results indicated the presence of a hydrogen bond between H hydroxyl (C7) of AST and O2 of C9.A and O (C ring), which can form a hydrogen bond to both N2 of G10.A and N2 of G16.B (Figure 7). The binding energy of AST (*E*_total_ −289.30) was found to be higher than that of kaempferol (*E*_total_ −238.29) when docking with the same method/form and this result could be due to the fact that kaempferol only can form a hydrogen bond between O (C ring) and N2 of G10.A and N2 of G16.B (figure not shown).

These docking data are compatible with our FTIR results that showed intercalation of AST into DNA (mainly with GC base pairs) stabilized by hydrogen bonds.

## 3. Experimental Section

### 3.1. Extraction Protocol

Fresh leaves (950 g) were freeze-dried and ground to obtain 320 g of dry material, which was extracted by decoction (1:20 p/v, 80 °C per 20 min) followed by filtration, rotary evaporation and freeze-drying to obtain 80 g of crude extract. Reverse phase-solid phase extraction was performed using the column Strata C18E 5 g/20 mL Giga Tubes (Phenomenex, USA). For each batch, 1 g of crude extract was diluted in 100 mL of distilled water. The cartridge was conditioned with acetone (25 mL) and washed with 5 mL of water before loading the sample (100 mL). It was then washed with 20 mL of water and eluted with 25 mL of ethyl acetate. After evaporation this eluent yielded the CaRP refined extract. The 80 g of crude extract yielded 4 g of CaRP that was stored in desiccators at room temperature in the dark until further use.

### 3.2. DPPH Assay

The free radical scavenging capacity of CaRP and of reference substances was determined using 2,2-diphenyl-1-picrylhydrazyl (DPPH) method according to Brand-Willians and co-workers [29]. Five concentrations of CaRP, ascorbic acid (AA), and Trolox in methanol were used to obtain the curves (Table 1). CaRP and standards (200 μL in the range of 2.66 to 7.09 μg mL^−1^) in methanol were added to 100 μL of DPPH (10 mg/100 mL MeOH) and allowed to stand for 30 min before absorbance was measured at 515 nm using a Molecular Devices (Sunnyvale, USA) spectrophotometer model Versamax Tunable Plate Reader. The DPPH solution (100 μL) and methanol (200 μL) were used as a negative control. This experiment was conducted in triplicate. Antioxidant activity was expressed as IC_50_ (inhibitory concentration in μg/mL necessary to reduce the absorbance of DPPH by 50% compared to the negative control). The lower the IC_50_, the higher was the antioxidant activity. Results were also expressed as AEAC (AA equivalent antioxidant capacity) or TEAC (Trolox equivalent antioxidant capacity) in grams and calculated as follows:

### 3.3. Strains and Media

The relevant genotypes of the yeast strain used in this work are given in Table 3. Media and solutions were prepared according to [30]. Complete medium (YPD) was used for routine growth of yeast cells and minimal medium (MM) was supplemented with the appropriate amino acids and uracil to yield SC (synthetic complete medium). Stationary (STAT) phase cultures with 2 × 10^8^ cells/mL were obtained after inoculation of an isolated colony into liquid YPD and after 72 h incubation at 30 °C with aeration by shaking. Cells were washed and diluted in saline (0.9% NaCl, pH 5.0) before all treatments.

### 3.4. Sensitivity Assay in Haploid Yeast to CaRP

The sensitivity of different yeast strains (STAT cells of wild type [WT] and of isogenic mutants) was evaluated on freshly prepared solid SC medium containing either no drug or 12 mg of CaRP. Cells were diluted in 1:10 steps in sterile saline and 5 μL of each dilution (suspensions containing from 10^7^ to 10^3^ cell/mL) was placed on agar surface of SC solid medium containing the appropriate compound. Cellular growth on SC was determined after 5 days incubation at 30 °C. Photos were taken using a Canon PowerShot G10 camera. Photos represent one of at least three independent experiments with similar results.

### 3.5. HPLC Analyses

#### 3.5.1. LC-UV DNA Binding of CaRP

This method was performed according to Zhou and co-workers [19] with slight modification. The centrifugal ultrafiltration was performed on an Eppendorf Refrigerated Centrifuge 5415R (Eppendorf, Germany). A 200 μL of CaRP (4 mg mL^−1^) and 300 μL of DNA solution (850 μg mL^−1^) were mixed in an Amicon centrifugal filter from Millipore (Bedford, MA, USA) with molecular weight cut-off of 10,000 Da and incubated at 37 °C in a thermo-mixer for 15 min. A blank sample was prepared with the addition of 300 μL of BPES buffer pH 7.0 (6 mM Na_2_HPO_4_, 2 mM NaH_2_PO_4_, 1 mM EDTA, 185 mM NaCl) instead of DNA. Samples were centrifuged at 4000 rpm for 30 min at 10° C, and the filtrates were analyzed by HPLC Shimadzu LC-20 Prominence series (Japan). Separations were achieved on a Kinetex C-18 column Phenomenex (100 × 3 mm I.D.; 2.6 μm 100 Å) with MeCN-water (10:90 to 90:10; 30 min). The flow-rate was 1.0 mL.min^−1^; UV absorbance was measured at 254 and 347 nm. The sample injection volume was set at 5 μL.

#### 3.5.2. LC-UV Microfractionation and Identification of the Fractions

The microfractionation of CaRP was performed according to Queiroz and co-workers [31]. CaRP was submitted to fractionation by LC performed on a Shimadzu LC-20AT Prominence with detector UV-Vis SPD-20A and collector FRC-10A (Japan). The separations were achieved on a Gemini C-18 column Phenomenex (150 × 10 mm I.D.; 5 μm 110Å) with MeCN-water (5:95 to 95:5; 1200 min). The sample injection volume was set at 250 μL (10 mg). The flow-rate was 0.2 mL min^−1^; UV absorbance was measured at 254 and 347 nm. Twenty fractions were collected for every peak with level >80,000 μV and width 10 s in plastic tubes (4 mL). After collection, all fractions were distributed in Eppendorf tubes (1.5 mL) and evaporated to dryness on a speedvac system (Eppendorf concentrator 5301, Germany). The fraction corresponding to peak 11 was identified using the LC analytical method described above. The semi-preparative chromatogram is shown in Figure 4.

Chromatographic preparation was repeated (about 50 injections) until obtaining 5 mg of dried mass of Fraction 11. The structural elucidation of this fraction was carried out by FTIR (KBr), HR MS/MS, ^1^H NMR and ^13^C NMR. A high resolution mass spectrum was recorded on a Waters ESI-Q-TOF mass spectrometer using the MassLynx V4.1 software package. ^1^H NMR and ^13^C NMR (APT) were recorded on a Bruker (300 MHz) NMR.

### 3.6. FTIR Analysis

Infrared spectra of dried fraction 11, obtained from the LC-microfractionation, were recorded on a Perkin Elmer Spectrum 400 FT-IR/FT-NIR spectrometer (Perkin Elmer, UK). Absorbance spectra were acquired at 4 cm^−1^ resolution and signal-averaged over 8 scans. For DNA interaction analysis, the fraction 11 (F11) was mixed with DNA separately in 1:2 ratio in BPES buffer pH 7.0 in sterile Eppendorf tubes; after a thorough mixing and overnight incubation at 37 °C, the samples were concentrated by speed-vac centrifugation (Eppendorf concentrator mod. 5301). DNA-F11 complexes were prepared and studied in solid-state using KBr pellet according to Usha and co-workers [22]. Spectra were processed using the SPECTRUM software from Perkin-Elmer. The de-convoluted parameters were set with a gamma value of 8.0 and a smoothing length of 90%.

### 3.7. Docking Study

The crystal structure of DNA-ligand complex was selected from Protein Data Bank PDB ID: 1D30 [32]. We docked AST onto the oligonucleotide extracted from the crystal structure. To determine the preferred binding sites on DNA and the binding energy of AST, docking studies were performed using HexServer [28]. HexServer is a fast Fourier transform (FFT)-based protein docking server to be powered by graphics processors. FFT-based approaches assume that the proteins (receptor) to be docked are rigid, but they sample densely all possible rigid-body orientations in the 6D search space [26]. The ligand structure was extracted from Pubchem (SDF: CID 5282102) file. To display docking results, we used UCSF Chimera 1.5.3 [33].

## 4. Conclusions

This study demonstrates that the *C. alata* leaf extract CaRP, obtained by reverse phase (C18)-solid phase extraction, exhibited potent DPPH free radical scavenging activity, was cytotoxic to 12 different *S. cerevisiae* DNA repair mutants and two mutants deficient in membrane transport. Using HPLC, three fractions of CaRP showed great affinity to bind DNA as revealed by a bio-fingerprinting chromatogram. Also, CaRP showed to be a good source of astragalin, which was shown, by FTIR and docking studies to interact with Gua-Cyt bases probably by intercalation stabilized mainly through H-bond formation.

## Figures and Tables

**Table 1:** IC_50_ (mean ± standard deviation), ascorbate and Trolox equivalent antioxidant capacity of *Cassia alata* leaves (CaRP) using 2,2-diphenyl-1-picrylhydrazyl (DPPH) assay.

Compound	IC_50_ (μg.mL^−1^)	AEAC (g)	TEAC (g)	linear range (μg.mL^−1^)	slope	intercept	Correlation coefficient (*r*)
ascorbic acid	3.99 ± 0.09	1.0000	0.8867	2.0–4.7	−12.131	98.414	0.9975
Trolox	4.50 ± 0.08	1.1278	1.0000	2.7–5.3	−12.275	105.230	0.9965
CaRP	2.25 ± 0.28	0.5639	0.5000	2.0–4.7	−14.733	83.457	0.9989

AEAC, ascorbate equivalent antioxidant capacity; TEAC, Trolox equivalent antioxidant capacity.

**Table 2:** The vibrational frequencies of major functional groups in free AST, free DNA and DNA-AST complexes.

Free AST, Free DNA and complexes	Observed changes in the vibrational frequency of the functional groups and bases (cm^−1^)							

	OH	C=O	NH	PO^−^_2_	Gua	Thy	Ade	Cyt
Free AST	3465–3426	1653	-	-	-	-	-	-
Free DNA	3469–3430	1707, 1649	3469–3430	1237, 1098	1707	1649	1602	1484
DNA-AST	3469–3435	1714, 1647	3469–3435	1236, 1094	1714	1647	1604	1488

**Table 3:** Yeast strains used.

strain	genotype	Protein lacking	source
BY4741	*Matα his3*Δ*1 leu2*Δ*0 lys2*Δ*0 ura 3*Δ*0*	none	EUROSCARF
*snm1*Δ	Like BY4741 except *snm1*Δ::KanMx4	5′-3′ exonuclease activity	EUROSCARF
*rad10*Δ	Like BY4741 except *rad10*Δ::KanMx4	Single-stranded DNA endonuclease (with Rad1p)	EUROSCARF
*rad1*Δ	Like BY4741 except *rad1*Δ::KanMx4	Single-stranded DNA endonuclease (with Rad10p)	EUROSCARF
*rad2*Δ	Like BY4741 except *rad2*Δ::KanMx4	Single-stranded DNA endonuclease	EUROSCARF
*xrs2*Δ	Like BY4741 except *xrs2*Δ::KanMx4	Protein required for DNA repair; Mre11 complex component	EUROSCARF
*rad5*Δ	Like BY4741 except *rad5*Δ::KanMx4	DNA helicase proposed to promote replication fork regression during postreplication repair	EUROSCARF
*rad52*Δ	Like BY4741 except *rad52*Δ::KanMx4	Protein that stimulates strand exchange by facilitating Rad51p binding to single-stranded DNA	EUROSCARF
*rad53*Δ	Like BY4741 except *rad53*Δ::KanMx4	Protein kinase, required for cell-cycle arrest in response to DNA damage	EUROSCARF
*rev1*Δ	Like BY4741 except *rev1*Δ::KanMx4	Deoxycytidyl transferase; involved in repair of abasic sites and adducted guanines in damaged DNA by translesion synthesis (TLS)	EUROSCARF
*rev7*Δ	Like BY4741 except *rev7*Δ::KanMx4	Accessory subunit of DNA polymerase zeta, involved in translesion synthesis during post-replication repair	EUROSCARF
*erg3*Δ	Like BY4741 except *erg3*Δ::KanMx4	C-5 sterol desaturase, catalyzes the introduction of a C-5(6) double bond into episterol, a precursor in ergosterol biosynthesis	EUROSCARF
*aus1*Δ	Like BY4741 except *aus1*Δ::KanMx4	Plasma membrane sterol transporter of the ATP-binding cassette family	EUROSCARF
*yor1*Δ	Like BY4741 except *yor1*Δ::KanMx4	Plasma membrane ATP-binding cassette (ABC) transporter, multidrug transporter mediates export of many different organic anions	EUROSCARF
SOD+	*Matα his3*Δ*1 leu2*Δ*0 trp1-289 ura3-52*	None	E. B. Gralla, Los Angeles
*sod2*Δ	Like SOD + except *sod2*::TRP1	Mn superoxide dismutase	E. B. Gralla, Los Angeles
*ctt1*Δ	Like SOD + except *ctt1*Δ::TRP1	cytosolic catalase T	E. B. Gralla, Los Angeles
*cta1*Δ	Like SOD + except *cta1*Δ::TRP1	catalase A present in peroxissomal matrix	E. B. Gralla, Los Angeles

## Abbreviations

CaRP: Reverse phase-solid phase extract from *Cassia alata* leaves
AST: astragalin
MRSA: Methicillin-resistant *Staphylococcus aureus*
